# Fever as a Clinical Presentation of Acute Hydrocephalus: Two Case Reports and a Review of Literature

**DOI:** 10.7759/cureus.19383

**Published:** 2021-11-08

**Authors:** Kenechukwu K Igbokwe, Ugochukwutumonye Odekpe, Reginald Ononye

**Affiliations:** 1 Neurological Surgery, Wellington Hospital Abuja, Abuja, NGA

**Keywords:** hyperpyrexia, shunt malfunction, ventriculoperitoneal shunt, acute hydrocephalus, fever

## Abstract

Fever is often associated with infectious, traumatic, or allergenic etiologies, a known consequence of the systemic inflammatory response. Its association with a rise in intracranial pressures (ICP) is an uncommon presentation. We herein describe two patients who developed acute hydrocephalus and presented with high-grade fever. This febrile component was resistant to conventional antipyretics and was only relieved by cerebrospinal fluid (CSF) diversion.

## Introduction

Fever is one of the most common clinical presentations encountered by physicians globally. It is a symptom and one of the most reliable signs of illness. Fever is defined as an elevation of body temperature due to the adjustment of the hypothalamic pituitary set point. Although variations in body temperature exist, the core body temperature is maintained at around 37°C (98.6°F). In adults, an oral body temperature reading of 37.2°C (98.9°F) and higher in the mornings or 37.7°C and higher in the evenings is considered to be fever. However, in children, rectal temperatures are the standard of care, and a rectal temperature greater than 38°C (100.4°F) is considered a fever [1].

Hydrocephalus is the accumulation of cerebrospinal fluid (CSF) in the ventricles of the brain as a result of a dynamic imbalance between the formation and absorption of cerebrospinal fluid. It is associated with ventricular dilatation and/or enlargement of the subarachnoid space [2]. The etiologies of hydrocephalus could be congenital or acquired. Acquired hydrocephalus can be caused by brain tumors, nontraumatic intracranial bleeding, meningitis, brain abscess, and head injury. Congenital hydrocephalus, on the other hand, is the second most common congenital brain malformation [3] and can be caused by neural tube defects, arachnoid cysts, Dandy-Walker syndrome, and Arnold-Chiari malformations, among others [4]. Diversion of accumulated CSF effectively treats hydrocephalus, and the standard for this is the insertion of a ventriculoperitoneal (VP) shunt [5]. This procedure aims to relieve the increased intracranial pressure (ICP) and improve symptoms; however, shunt malfunctions can arise and eventually lead to elevations in intracranial pressure, which can abruptly cause deterioration in patient condition.

The association between fever and hydrocephalus was first reported by Talman et al. in 1988 [6]. They observed the onset of fever and autonomic dysfunction in two patients with acute hydrocephalus secondary to shunt malfunction.

In this case series, we illustrate this observation in two patients with fever as a clinical presentation of acute hydrocephalus in Wellington Hospital Abuja. We aim to highlight the underreported association between fever and hydrocephalus as well as the diagnostic dilemma in arriving at the clinical diagnosis.

## Case presentation

Case 1

A 50-year-old female presented on referral with complaints of blurring of vision on the left eye, dizziness, headache, and generalized heaviness of the body of six months' duration. She was managed conservatively at another hospital, and with consults sent to the neurologist and physiotherapist, her symptoms were largely unchanged. She was scheduled for neuroimaging but was unable to get one due to financial constraints. Her past medical history was significant for uncontrolled hypertension. Two weeks before she presented to our facility, she was admitted to the emergency room at the general hospital on account of a sudden onset of severe headaches and transient loss of consciousness. She was advised on a cerebral angiogram that showed a terminal internal carotid artery (ICA) aneurysm prompting her referral to our facility and global hyperreflexia and hypertonia. On visual examination, she had perception of light in both eyes.

On examination, her general condition was stable, with a Glasgow Coma Scale (GCS) score of 15/15 and absent meningeal signs. Cranial nerve examination showed equal and briskly reactive pupils bilaterally, reduced visual acuity in the left eye (6/18), and a left lateral conjugate gaze palsy. The patient had normal tones and reflexes globally and no cerebellar signs. She was counseled on the findings and diagnosis, admitted into our facility, and worked up for a clipping of the terminal ICA aneurysm. Laboratory workup revealed no abnormalities (Table 1).

**Table 1:** Summary of the laboratory results for case 1.

	Pre-aneurysm clipping workup	Post shunt malfunction
CBC	Hb: 13.3 g/dL, WBC: 5.2 × 10^3^ cells/mm^3^, platelets: 180,000 cells/mm^3^	Hb: 12.2 g/dL, WBC: 12.3 × 10^3 ^cells/mm^3^ (granulocytes: 83.9%), platelets: 165,000 cells/mm^3^
Electrolytes, urea, and creatinine	Na: 139 mEq/L, K: 3.6 mEq/L, Cl: 97 mEq/L; urea: 43 mg/dL; creatinine: 0.9 mg/dL	Na: 132 mEq/L, K: 3.45 mEq/L, Cl: 100 mEq/L; urea: 30 mg/dL; creatinine: 1.3 mg/dL
Clotting profile	PT: 14 seconds, aPTT: 25 seconds, INR: 1.1	
CSF analysis		WBC: 4 cells/mm^3^ (lymphocytic predominance: 95%), RBC: 3 cells/mm^3^, glucose: 65 mg/dL, protein: 30 mg/dL
Urinalysis		pH: 7, specific gravity: 1.025, others: nil
Blood cultures (after 72 hours of incubation)		Yielded no growth
CSF microscopy and culture		Gram staining: no organism seen, culture (after 48 hours of incubation): yielded no growth

**Figure 1:**
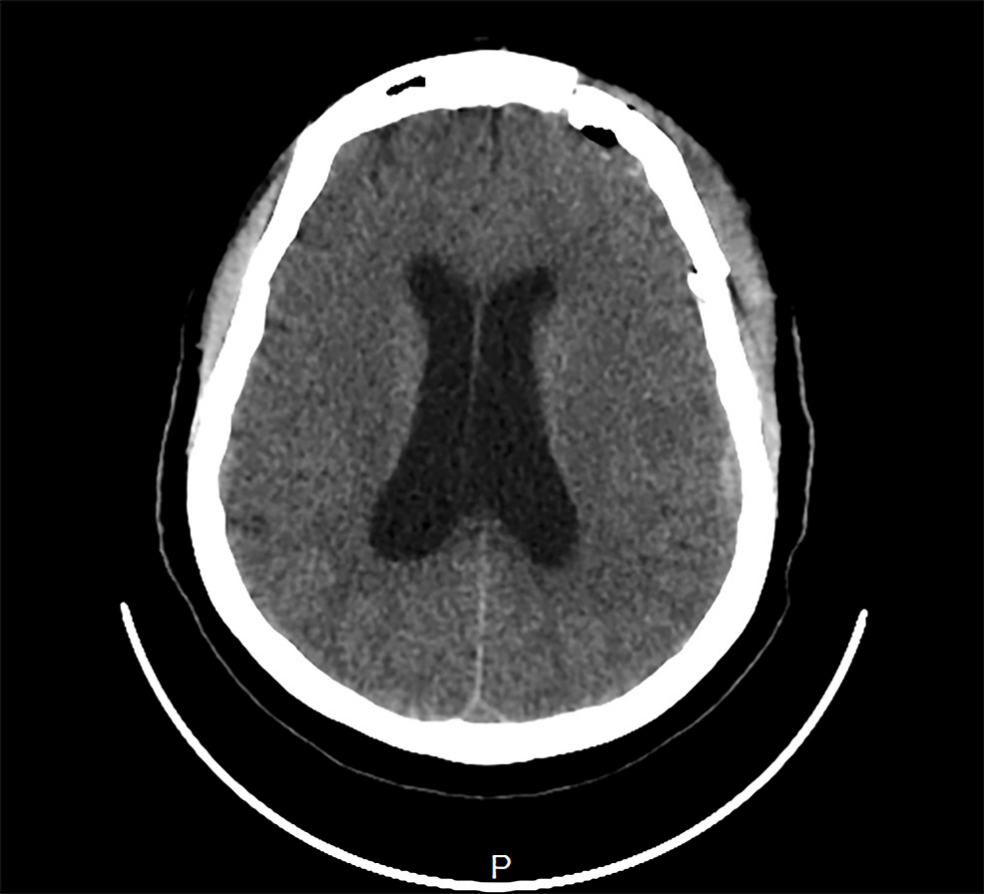
Dilated lateral ventricles.

**Figure 2:**
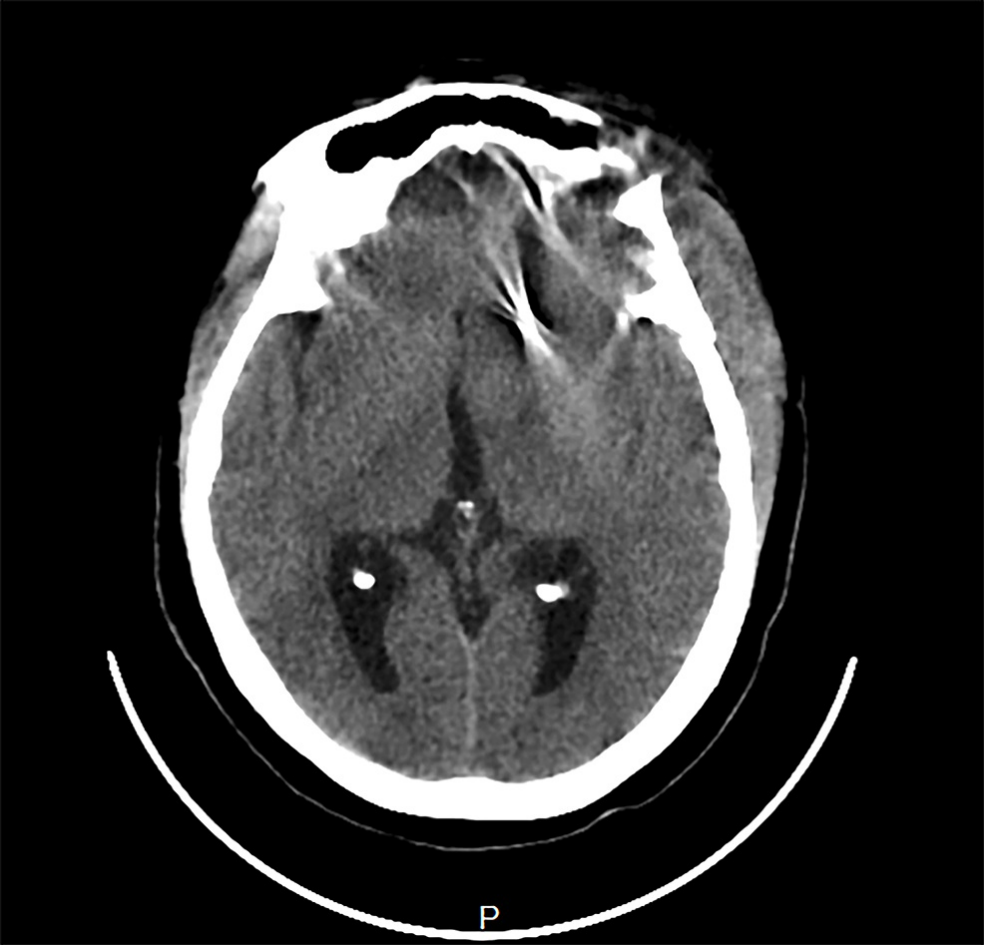
The dilated occipital horns of the lateral ventricles are demonstrated. The hyperdense shadow of the aneurysm clip is also observed in the left cerebral hemisphere.

Intraoperative findings confirmed a left ophthalmic segment ICA aneurysm, which compressed and flattened the left optic nerve, as well as extensive arachnoid adhesions in the region of the aneurysm. Postoperative recovery was in the intensive care unit (ICU). On the first day postoperatively, the patient's level of consciousness was observed to be declining (GCS dipped from 15/15 to 10/15), necessitating an urgent brain computed tomography (CT), which revealed a subdural and subgaleal hematoma, which was evacuated. Her recovery was good and sustained, and she was subsequently discharged from the ICU. In the second week postoperatively, she had several bouts of projectile vomiting and irrational behavior characterized by refusal of feeds and aggression toward the nursing care. The suspicion of a raised intracranial pressure was confirmed by a non-contrast brain computed tomography (CT) scan showing dilated lateral ventricles (Figures 1, 2). This was promptly treated with a ventriculoperitoneal (VP) shunt.

Her clinical condition improved following the VP shunt revision. The shunt position and efficacy were confirmed via a check brain CT scan. Her clinical state yet again deteriorated on the eighth day post-VP shunt insertion, evidenced by a high-grade fever of up to 40.6°C (105.08°F), dropping level of consciousness (GCS score: 12/15; eye response (E): 3, verbal response (V): 4, motor response (M): 5), and irrational behavior. She was tachycardic and tachypneic, with oxygen saturations between 94% and 96%. Samples were taken for urgent laboratory tests, including blood and urine cultures. Table 1 presents the values of the repeat laboratory evaluation.

**Figure 3:**
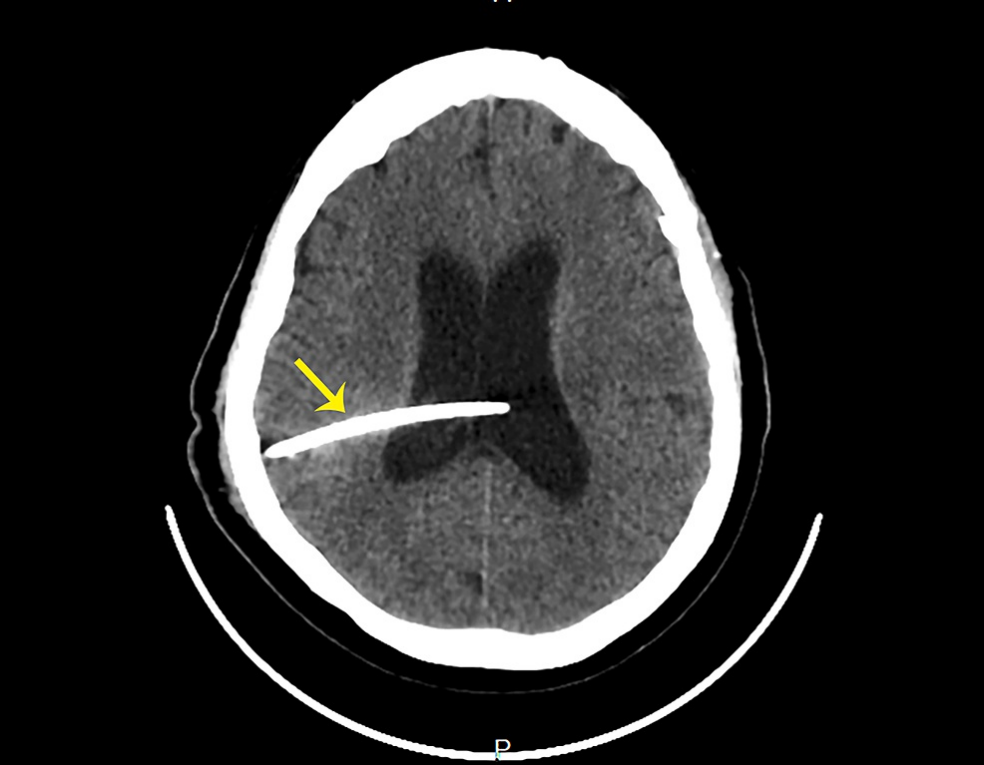
Ventriculoperitoneal catheter (yellow arrow) in situ traversing the right cerebral hemisphere into the lateral ventricles.

A non-contrast brain CT revealed a shunt in the ventricular cavity and dilatation of the lateral ventricles and the third ventricle (Figure 3).

She was booked for an emergency shunt revision surgery. Intraoperative findings confirmed high CSF pressure and shunt obstruction. The opening pressure on the shunt was 26 cmH_2_O. Twenty-four hours after shunt revision surgery, her body temperature and other vital signs returned to normal, and her altered consciousness improved.

Case 2

**Figure 4:**
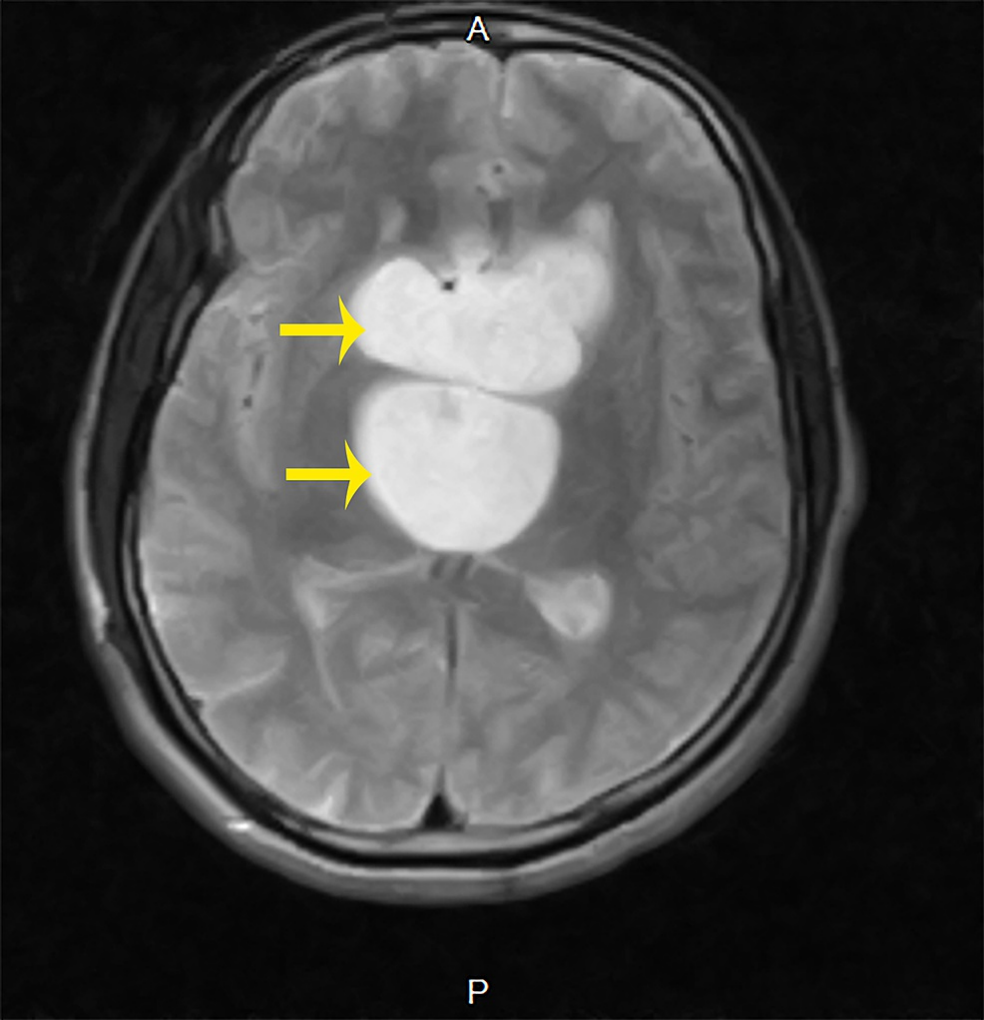
T2-weighted brain MRI showing contrast-enhancing space occupying the lesion in the suprasellar compartment.

**Figure 5:**
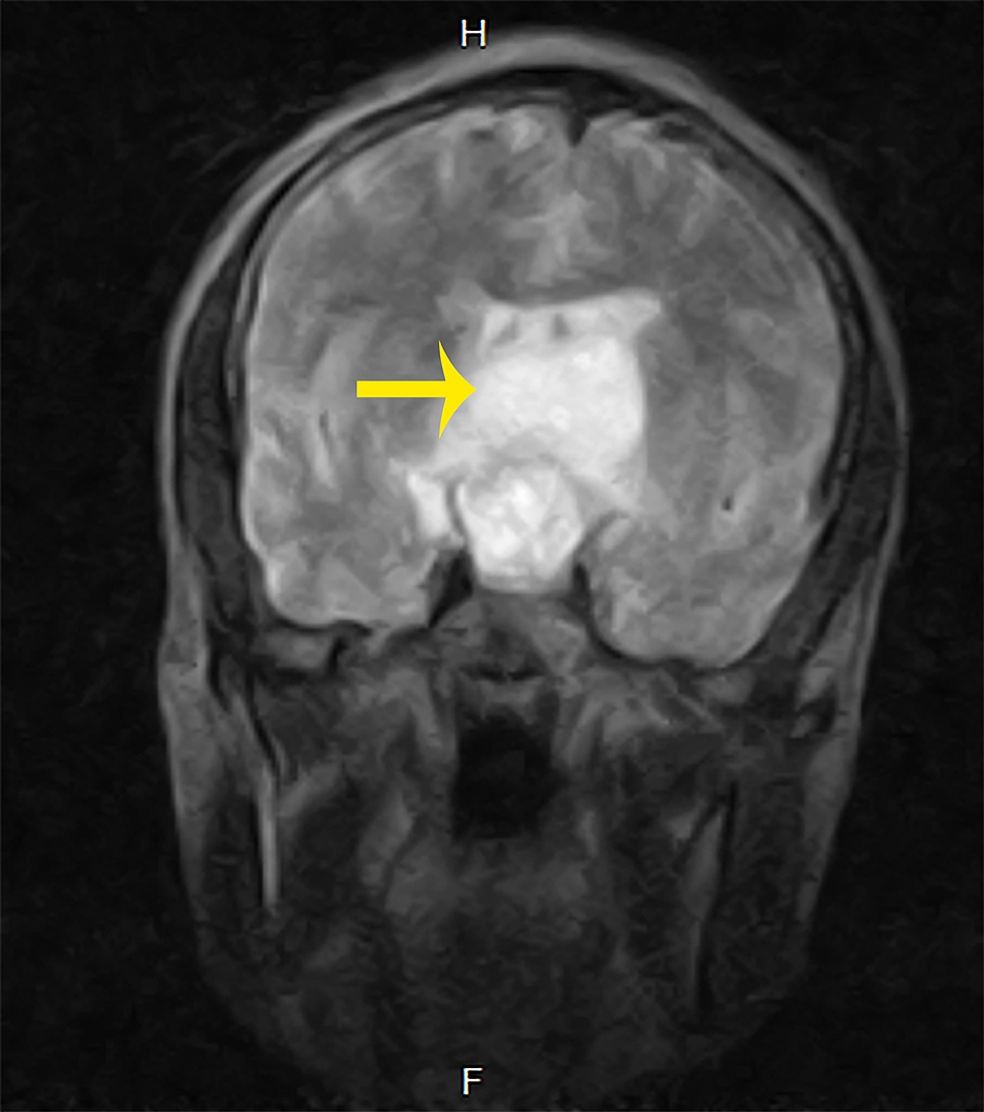
T2-weighted brain MRI showing a suprasellar mass.

A 31-year-old male with three months history of sudden onset of headaches and recurrent seizures was referred to our facility on account of a non-homogenous contrast-enhancing large suprasellar mass with obstructive hydrocephalus seen on contrasted brain magnetic resonance imaging (MRI) (Figures 4, 5).

The neurological examination results were within normal limits. A treatment plan was staged for tumor excision, which involved an initial VP shunt insertion to relieve symptoms and divert CSF and a subsequent tumor excision surgery. Following shunt insertion, the presenting symptoms of headaches resolved while he was maintained on anticonvulsant medications. About two months after VP shunt insertion, he presented to our center with episodes of confusion and drowsiness, urinary incontinence, and a high-grade fever of 38.7°C (101.6°F). His GCS score was 11/15 (E: 2, V: 4, M: 5), and his random blood sugar was 7.2 mmol/L. Both pupils were 4 mm and sluggishly reactive, and his muscle tones were normal. Samples for blood and urine cultures, complete blood count, and electrolyte evaluations were drawn, and he had a brain CT scan done. The head CT scan demonstrated marked enlargement of the lateral and third ventricles, which was suggestive of possible malfunction. His laboratory blood workup results (Table 2) were within normal limits.

**Table 2:** Laboratory results for case 2.

	Post shunt malfunction
CBC	Hb: 15 g/dL, WBC: 18 × 10^3^ cells/mm^3^ (granulocytes: 81.2%), platelets: 240,000 cells/mm^3^
Electrolytes, urea, and creatinine	Na: 145 mEq/L, K: 4.8 mEq/L, Cl: 105 mEq/L; urea: 52 mg/dL; creatinine: 1.4 mg/dL
Urinalysis	pH: 6, specific gravity: 1.030, others: nil
CSF microscopy and culture	Gram staining: no organism seen, culture: no growth after 48 hours of incubation
Blood culture	No growth after 48 hours of incubation

He was taken to the operating room for revision of the ventriculoperitoneal shunt. After the shunt was revised, there was a resolution of his fever and tachycardia. Urinary incontinence also improved in the following days. He, however, had residual confusion postoperatively that necessitated commencement on antipsychotic medication (haloperidol). Blood and urine cultures were not consistent with an underlying infective process. Subsequently, he showed steady neurological improvement and was booked for a definitive procedure to excise the suprasellar mass.

## Discussion

Acute intracranial hypertension is a well-recognized cause of autonomic disturbance. In our reported cases, however, fever was the predominant clinical sign outside the usual signs of raised ICP. Intracranial hypertension was reported to be associated with the Cushing triad, which consists of hypertension, bradycardia, and decreased respiratory drive [6]. This phenomenon has also been shown to cause alterations in the circadian rhythm of the body temperature [7]. However, disturbances in the body temperature were not reported until Talman et al. made their report of two cases that presented with a hyperpyrexic syndrome that responded immediately and fully to the treatment of underlying hydrocephalus [6]. Talman et al. and some other authors [6,8,9] proposed the possible mechanism for this occurrence - an abnormal response of the hypothalamic nuclei responsible for thermoregulation and other endocrine homeostatic functions, as well as a disruption of the communicating periventricular neuronal networks. This disruption could also account for the irrational behavior seen in both patients in this report. We suppose that third ventricular dilatation can mimic the natural febrile response by exerting pressure effects on the hypothalamic nuclei (anterior nuclei, which is responsible for cooling the body, and posterior nuclei, which is responsible for elevating body temperature).

Our first patient developed features that mimic an acute infective process - sustained hyperpyrexia, vomiting, and irrational behavior. She was tachycardic and tachypneic, but her blood pressure was within the normal range as she was commenced on antihypertensive. Clinical assessment of the subcutaneous path of the shunt for any signs of inflammation or an inability to palpate the shunt revealed no abnormality. Samples for complete blood count, CSF analysis, urinalysis, urine microscopy, and blood culture were unremarkable. CBC revealed leukocytosis with granulocyte predominance (83%), mild hyponatremia, and mild hypokalemia. The results of the CSF, urine, and blood cultures returned negative. Her symptoms resolved after VP shunt insertion surgery. It is important to note, however, that the first episode in this patient of acute elevation in ICP postsurgical intervention (clipping of aneurysm) did not present with fever. This is in contrast to the second episode that presented with a high-grade fever among other signs of acute hydrocephalus. This emphasizes that the absence of fever does not preclude the diagnosis of acute hydrocephalus.

The second patient initially had VP shunt insertion to treat obstructive hydrocephalus secondary to a suprasellar mass. He had been discharged from the hospital two months prior to the onset of symptoms. He was yet to have a tumor excision surgery when he developed symptoms of severely throbbing headaches, confusion, urinary incontinence, and hyperpyrexia of 39.6°C (103.28°F). His GCS score was 11/15, his pupils were sluggishly reactive, and there was no focal neurological deficit that localizes the lesion. Head CT scan showed marked enlargement of the lateral and third ventricles, which was suggestive of possible shunt obstruction. CSF, blood, and urine culture reports were not consistent with an underlying infective process.

Both patients discussed above developed an acquired type of acute hydrocephalus in the postoperative period. This was heralded by symptoms of raised intracranial pressure and pyrexia that were unresponsive to the conventional tepid sponging, parenteral paracetamol dosage at 600-900 mg, and suppository diclofenac. This febrile syndrome was subsequently corrected by surgical reconstruction of a CSF diversion in these patients by way of a VP shunt.

Several diagnostic possibilities other than hydrocephalus could account for a similar syndrome in these patients (see Table 2), particularly an infectious etiology, which was unlikely, as evidenced by the negative culture results. The immediate resolution of the fever upon correction of the hydrocephalus is the major indicator to that acute hydrocephalus was the more likely etiology of the febrile response.

## Conclusions

We have discussed an association of pyrexia and acute hydrocephalus in two postsurgical patients in the field of neurosurgery. This may present a diagnostic challenge as fever in this kind of patient would firstly suggest an infective origin. Diagnosis of acute hydrocephalus is aided by a non-contrast brain CT to initiate treatment and prevent further deterioration of the patients.

A febrile component in acute hydrocephalus is not a common presentation. Further scientific research is therefore required to underscore the effect of hydrocephalus on the hypothalamus and understand its pathophysiology.
